# Hypermethylation of *SOX1* and *HOXA9* Genes Is Associated with Clinicopathologic Characteristics of Non-Small Cell Lung Cancer Patients

**DOI:** 10.3390/cimb47060397

**Published:** 2025-05-26

**Authors:** Milica Kontić, Mihailo Stjepanović, Filip Marković

**Affiliations:** 1 Pulmonology Clinic, University Clinical Center of Serbia, 11000 Belgrade, Serbia; 2 School of Medicine, University of Belgrade, 11000 Belgrade, Serbia

**Keywords:** methylation, non-small cell lung cancer, SOX1, HOXA9, clinicopathologic characteristics

## Abstract

DNA methylation changes, especially hypermethylation of *SOX1* and HOXA9, may serve as biomarkers for diagnosis and prognosis in non-small cell lung carcinoma (NSCLC). This study analyzed the methylation status of *SOX1* and *HOXA9* in 63 primary NSCLC tumor samples, corresponding normal lung tissues, and circulating blood, using bisulfite pyrosequencing. The relationship between methylation patterns and clinicopathologic features was also explored. *SOX1* and *HOXA9* promoter methylation levels were significantly higher in tumor tissues compared to normal lung tissues and blood samples. Histological subtypes influenced methylation patterns, with squamous cell carcinomas (SCC) showing higher hypermethylation rates at both loci compared to other NSCLC subtypes. *HOXA9* hypermethylation was associated with advanced tumor stage (stages II and III). Gender and smoking status did not correlate with methylation status. These findings highlight the cancer-specific nature of *SOX1* and HOXA9 hypermethylation in NSCLC. Further investigation into demographic and molecular factors influencing methylation could enhance the clinical utility of *SOX1* and *HOXA9* in NSCLC diagnosis and management.

## 1. Introduction

Lung cancer remains the leading cause of cancer-related death worldwide, with non-small cell lung cancer (NSCLC) accounting for approximately 85% of cases [1]. Despite advances in cancer diagnosis and treatment over the previous years, patients diagnosed with metastatic disease (stage IV) had a 1-year survival rate of just 15–19% compared with 81–85% for stage I [2]. It seems as though poor prognosis is associated with diagnosing the disease in its later stages, and that early diagnosis may lead to better outcomes. However, even patients with early-stage NSCLC who undergo complete surgical resection face a high risk of recurrence, with a 5-year relapse rate of up to 50% [3].

Identifying reliable biomarkers with diagnostic and prognostic value is crucial for improving early detection, predicting outcomes, and guiding therapeutic decisions in NSCLC.

The epigenetic control of gene expression plays an important role in carcinogenesis. The aberrant methylation of CpG dinucleotides is a commonly observed epigenetic modification in human cancer, and it appears to be an alternative mechanism of gene silencing in human tumorigenesis [4].

Several studies have established that DNA methylation changes are associated with clinical outcomes in NSCLC, highlighting their potential as prognostic and predictive markers as well as their utility in early diagnosis [5,6,7,8]. By translating these findings into clinical practice, new avenues for screening, early diagnosis, and treatment stratification may be realized.

Tumor suppressor genes (TSGs) are potentially useful markers for LC detection, progression and treatment target [4,9]. Several tumor suppressor genes have been reported as aberrantly methylated in lung tumors [10]. We have examined the literature to identify genes most frequently hypermethylated in NSCLC, which led us to the investigation of the genes *HOXA9* and *SOX1* that have shown potential as diagnostic and prognostic biomarkers in NSCLC [11,12].

The aim of this study was to determine whether these methylation changes are specific to NSCLC by using bisulfite pyrosequencing, and to test whether these changes are detectable in patients’ blood samples. Additionally, we sought to examine the correlation between the promoter hypermethylation of these genes and their clinicopathologic features.

## 2. Material and Methods

Primary tumor samples (n = 63), corresponding nonmalignant lung tissues (n = 63), and matching blood samples (n = 51) were obtained from patients with NSCLC who had been treated in 2009 with curative surgical resection at the Clinic for Pulmonology, University Clinical Center of Serbia. The samples were collected during surgery and immediately snap-frozen for research purposes. Patients had no neoadjuvant chemotherapy or radiotherapy and patients with large cell-carcinoma were not included in the study.

The study was approved by the institution’s ethics committee, and informed consent was obtained from all study participants.

Demographic data were obtained through patient interview, and clinical information was derived from chart review.

Blood samples (5 mL) were collected using EDTA vacutainers and stored at −20 °C. DNA extraction from whole blood samples was performed using the method previously described [8]. Briefly, blood cells and platelets were lysed by adding equal volumes of lysis buffer (0.32 M sucrose, 10 mM Tris-HCl pH 7.5, 5 mM MgCl_2_ and 1% Triton X-100). The lysate underwent centrifugation (3000 rpm for 10 min). Following the removal of supernatant, the pellet was suspended in 3 mL buffer (10 mM Tris-HCl, 0.4 M NaCl, 2 mM EDTA, and 25 μL proteinase K), followed by the addition of SDS (0.7% final concentration). After overnight incubation at 37 °C, 1 mL of 6 M NaCl was added, and the proteins were pelleted by centrifugation. Supernatant containing DNA was transferred into fresh tubes and centrifuged (4000 rpm for 10 min). The supernatant was transferred into new microcentrifuge tubes and an equal volume of isopropanol was added. DNA that became visible was transferred and washed in 1 mL 70% ethanol, air-dried, resuspended in distilled water, analyzed for quality by electrophoresis on agarose gel and quantified by using a Nanodrop spectrophotometer (ThermoScientific Inc., Wilmington, DE, USA). The isolation of DNA from fresh frozen tumors was performed as previously described [5]. The subsequent laboratory research was carried out at the Masonic Cancer Center, University of Minnesota, USA. The primer sequences and PCR conditions have been described previously [9,13,14,15]. Amplicons were confirmed via agarose electrophoresis, and methylation percentages were calculated using PyroMark software (Qiagen, Hilden, Germany) version 2.0.8.

The detection of DNA methylation was undertaken based on a treatment of genomic DNA with sodium bisulfite, which converts unmethylated cytosines to uracil, while methylated cytosines stay unaltered. Bisulfite-modified DNA as per manufacturer’s protocol (Zymo Research, Irvine, CA, USA) was then subjected to strand-specific polymerase chain reaction to generate templates for pyrosequencing. In our study, the sequencing depth and coverage were carefully monitored to meet the standards required for methylation analysis. Specifically, we achieved an average sequencing depth of approximately 500–1000 reads per CpG site, with a minimum depth of 200 reads for inclusion in the analysis. This ensured sufficient coverage to accurately detect methylation patterns.

### Statistical Analysis

The statistical analyses included calculations for each CpG site and the average across multiple CpG sites for each gene. Since methylation values were not normally distributed, the Wilcoxon signed-rank test was applied to the average methylation across all CpG sites within a gene to assess differences between tumor and normal tissues. When the analysis was repeated using individual CpG sites instead of gene-level averages, the results remained consistent. Tumors were classified as hypermethylated if the average methylation across different CpG sites for a specific gene exceeded the corresponding average methylation in normal tissues by more than three standard deviations, as previously described [9]. Comparisons of blood methylation levels between patients with hypermethylated tumors and those without were performed using the Wilcoxon rank-sum test.

All analyses were performed using the R package version 0.5.5.

## 3. Results

The correlations of clinicopathologic characteristics with the hypermethylation of tumor profiles of the NSCLC patients are summarized in Table 1.

Stage-wise analysis showed that *HOXA9* hypermethylation was more prevalent in stage II and III tumors compared to stage I (*p* = 0.03). No significant stage-wise difference was observed for *SOX1* methylation (*p* = 0.24). In squamous cell lung cancer, hypermethylation in genes *SOX1* (*p* = 0.05) and *HOXA9* (*p* = 0.01) is more frequent than in other histological types of NSCLC.

Patients with stage II and III showed more frequent hypermethylation in gene HOXA9.

There were no significant associations between the methylation status of *SOX1* and HOXA9 and patient gender or smoking status (Table 1).

The numbers of CpG islands withing the promoters of genes *SOX1* and *HOXA9* were 4 and 11, respectively (Table 2).

The methylation levels of *SOX1* and *HOXA9* were significantly higher in NSCLC tissue samples compared to normal lung tissues and blood (Table 3 and Table 4).

Intraclass correlation coefficients (ICCs) were calculated for both genes using ANOVA, for all tissue types. High values mean great methylation variability between patients.

Higher values were detected in tumors compared to normal lung tissue and blood samples (Table 5). This is the result of great differences in methylation between tumors, as some tumors were hypermethylated and others were not.

The following tables provide a comprehensive overview of DNA methylation patterns for *SOX1* and *HOXA9*—Table 6 presents the mean methylation values across all CpG islands in promoter regions, Table 7 summarizes the proportion of hypermethylation across different tissue types, and Table 8 details the total number of patients exhibiting hypermethylation in tumor DNA.

**Figure 1:**
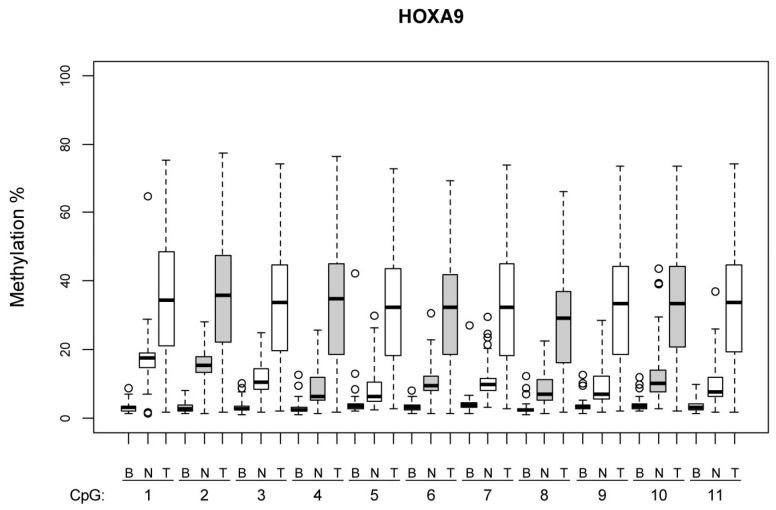
Graphical representation in boxlots. Methylation in every single CpG island inside promotor gene HOXA9 based on tissue type (tumor—T, normal lung tissue—N, blood—B). Methylation in tumors was higher than in normal lung tissue. Methylation in tumors was higher than in blood samples. Normal lung tissue had a higher percentage of methylation than in matching blood samples.

**Figure 2:**
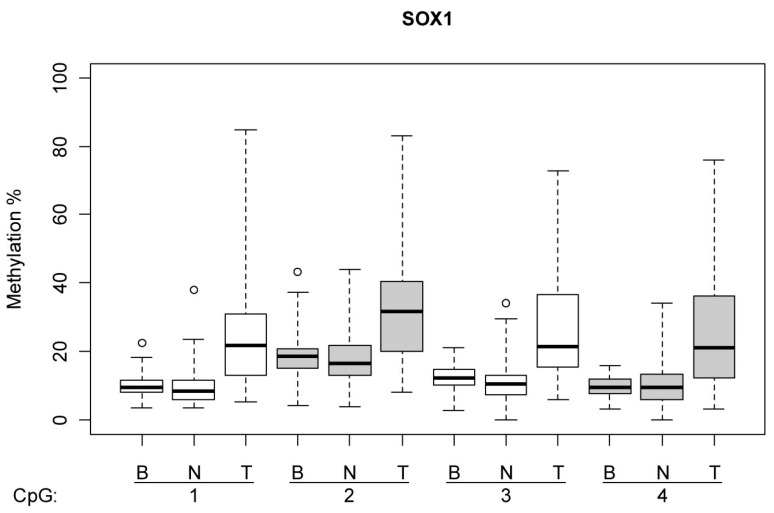
Graphical representation in BoxPlot form. Methylation in every single CpG island inside promotor gene SOX1 based on tissue type (tumor—T, normal lung tissue—N, blood—B). Methylation values in tumors were higher than in normal lung tissue. Methylation levels in tumors were higher than in blood samples. Normal lung tissue did not have higher percentages of methylation than matching blood samples.

**Figure 3:**
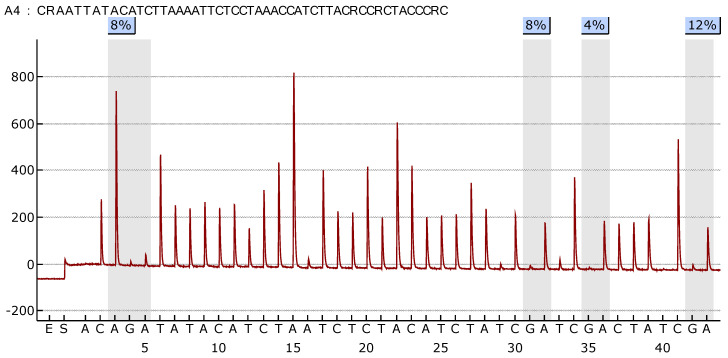

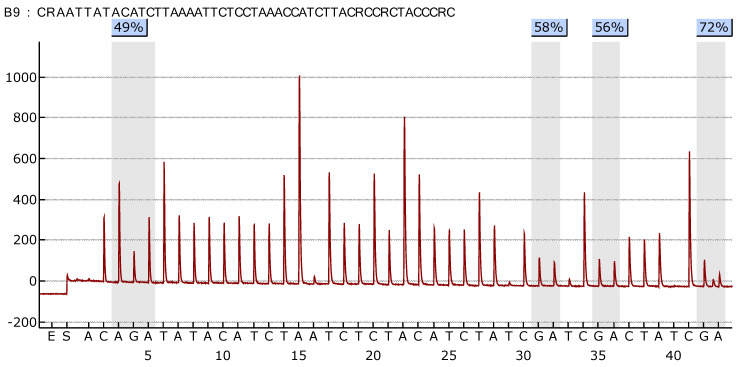
SOX1 gene—difference in the methylation profile of this gene between normal lung tissue (**up**) and tumor (**down**) in the same patient.

Figure 1 and Figure 2 illustrate the methylation levels at individual CpG islands within the *HOXA9* and *SOX1* promoters, respectively, across different tissue types (tumor, normal lung tissue, and blood), highlighting the tissue-specific differences in methylation patterns. Figure 3 illustrate the difference in the methylation profile of *SOX1* gene between normal lung and tumor tissue in the same patient.

## 4. Discussion

The elevated methylation levels of *SOX1* and *HOXA9* in tumor samples compared to normal lung tissue and blood samples indicate that these epigenetic alterations are specific to NSCLC. However, the lack of significant methylation differences in blood samples compared to tumor tissue suggests that blood-based assays might not be an indicator of hypermethylation in tumor tissue, and cannot serve as a tool for the early diagnosis of lung cancer. This observation is in accordance with previous studies comparing the hypermethylation of genes in tumor tissues and matched blood samples using bisulfite pyrosequencing in patients with resectable NSCLC [11,16,17]. On the other hand, Wen et al. reported a sensitivity of 75.0% and a specificity of 98.0% when detecting methylated *HOXA9* in blood samples of NSCLC patients with methylated *HOXA9* tumors [18]. The advanced stage and histology-specific nature of their cohort, as well as its size, may explain the discrepancy with our findings. Methylation differences in tumor and normal lung tissue were also evident [18].

Although we evaluated the significance of *SOX1* and *HOXA9* hypermethylation individually, we did not assess the combined effects of the simultaneous hypermethylation of both genes due to the limited sample size. Future studies with larger cohorts are warranted to explore the potential synergistic impacts of co-methylation on patient outcomes.

The HOX gene family, which includes *HOXA9*, and the *SRY* box gene family, which includes the *SOX1* gene, are crucial for normal embryogenesis [11,19]. The hypermethylation of both *HOXA9* and *SOX1* genes has been reported in various malignancies including NSCLC, with the *SOX1* gene being one of the most frequently hypermethylated genes [11]. The exact roles and mechanisms by which these genes impact carcinogenesis are not yet fully understood. Some data derived from preclinical and experimental studies suggest that the reduced expression of *SOX1* and HOXA9 leads to the disruption of normal processes of cell migration in NSCLC [20,21].

*SOX1* suppresses Rac1 activity, a key member of the Rho family of GTPases that regulates actin cytoskeletal remodeling and membrane protrusion formation—critical steps in cancer cell motility. The loss of SOX1, as a result of promoter hypermethylation, enhances Rac1-mediated actin dynamics, leading to increased membrane protrusions and elevated cell migration. Since increased cell motility and invasion are key drivers of metastasis, the methylation-induced silencing of *SOX1* likely facilitates tumor dissemination, and is thus associated with poorer clinical outcomes in NSCLC patients [22].

The role of *SOX1* and *HOXA9* methylation in prognosis remains a subject of ongoing investigation. Hwang et al. found that the hypermethylation of *HOXA9* was associated with disease recurrence in early-stage NSCLC patients [20]. According to Ben et al., the hypermethylation of *HOXA9* was associated with worse progression free and overall survival among advanced-stage NSCLC patients undergoing PD-1/PD-L1 inhibitor therapy [22]. In a meta-analysis, Cai et al. found that the hypermethylation of HOXA9 was associated with poor overall survival among patients with solid malignancies including NSCLC [19]. This contrasts with the more recent findings of Vicente at al., that the hypermethylation of *HOXA9* is not an independent prognostic biomarker of cancer-specific survival among patients with lung adenocarcinoma [23]. These discrepancies may stem from differences in cohort size, with characteristics including disease stage, histological types, and potential publication bias favoring positive findings [19,23,24]. Vicente et al. also found the that hypermethylation of *HOXA9* was associated with earlier stages of the disease, as was the case with our cohort, which was smaller in comparison to theirs [23]. This finding, along with that of Hwang et al., that the hypermethylation of *HOXA9* was associated with the disease recurrence of early-stage NSCLC, may serve to guide further trials of implementing hypermethylation in *HOXA9* detection in blood-based assays for minimally invasive screening and monitoring programs. One study found that a methylation panel of six genes including HOXA9 has shown potential for use as a biomarker for early NSCLC detection [17].

In our previous work, we found that *SOX1* promoter hypermethylation in NSCLC tumors was significantly associated with inferior survival [11].

This highlights the potential of *SOX1* and *HOXA9* methylation as prognostic biomarkers, emphasizing the need for further studies to validate their clinical relevance and inform risk stratification.

Ever since DNA methylation changes have been associated with carcinogenesis, efforts have been made to investigate their prognostic capabilities as well as clinical and pathological characteristics in order to inform optimal patient selection. Data regarding the clinical and pathological characteristics of NSCLC patients with hypermethylated *SOX1* and *HOXA9* remain scarce.

Our analysis has revealed that histology, specifically squamous cell carcinoma, is significantly associated with the hypermethylation of both *SOX1* and *HOXA9*.

While investigating the methylation status of another group of commonly methylated genes in early NSCLC that have shown prognostic capabilities (*RASSF1A*, *CDH13*, *MGMT*, *ESR1* and *DAPK*), several groups of authors have reported conflicting results. While some reported that the methylation of *CDH13* was associated with lung adenocarcinoma, others found no difference among major histological types of NSCLC [9,25,26]. Our findings may be influenced by the size of our cohort, and reflect the high prevalence of squamous cell carcinoma in our study.

Additionally, *HOXA9* hypermethylation was more prevalent in patients with stage II and III tumors when compared to stage I, suggesting its potential role in tumor progression and aggressiveness. Considering the mentioned association of the hypermethylation of genes in the tumor tissue but not the normal lung tissue, the prevalence of hypermediated tumor suppressor genes could be expected to rise in later stages of NSCLC [6]. This is in contrast with the recent finding of Du et al., who found that *HOXA9* hypermethylation was not significantly associated with advanced NSCLC stage when comparing patients in stage IA to the rest of the cohort [26]. In their study, there was a much higher prevalence of patients with stage I disease and adenocarcinoma in comparison to ours, which may explain this difference in results.

Gao et al. found that the number methylation-positive *SHOX2* and *RASSF1A* patients increased with clinicopathological stage of the disease, and the age of the patients affected by lung adenocarcinoma [6]. While the methylation of certain oncogenes, namely, *RASSF1A* was associated with gender in NSCLC patients, in our previous work among the patient cohort from our center, we did not find such an association [9]. These discrepancies warrant further investigation into the influence of demographic and lifestyle factors on gene methylation patterns in NSCLC, given their potential as prognostic biomarkers.

Although the current study did not directly assess downstream molecular signaling, prior evidence suggests that the hypermethylation-induced silencing of *SOX1* and *HOXA9* may impact key oncogenic pathways in NSCLC. *SOX1* has been shown to act as a tumor suppressor by antagonizing the Wnt/β-catenin signaling pathway. Specifically, *SOX1* can interact with β-catenin and prevent its transcriptional activity, thereby inhibiting Wnt target gene expression and limiting tumor progression [20]. The epigenetic silencing of *SOX1* through promoter hypermethylation, as observed in our cohort, may thus lead to the aberrant activation of the Wnt pathway, contributing to the increased proliferation and survival of cancer cells. On the other hand, *HOXA9* has been implicated in regulating Notch signaling, particularly in hematologic malignancies and some solid tumors. Aberrant *HOXA9* expression can modulate components of the Notch pathway, including HES1 and DLL4, and influence stemness, differentiation, and cell fate decisions [27,28]. While direct evidence in NSCLC is limited, the potential for epigenetically silenced *HOXA9* to disrupt these regulatory networks highlights a plausible mechanism underlying tumor progression. These interactions between epigenetic regulation and developmental signaling pathways represent important areas for future functional studies in lung cancer.

Several studies have shown that *PAX1* and *SOX1* are frequently hypermethylated in cervical neoplasia. Combined methylation analysis of these genes has been effectively used as a screening tool for cervical intraepithelial neoplasia (CIN) and cervical cancer, demonstrating good sensitivity and specificity [27,28]. In esophageal squamous cell carcinoma (ESCC), *PAX1* has also been identified as a hypermethylated gene. Methylation markers such as *PAX1, ZNF582*, and *SOX1* have been investigated as potential diagnostic biomarkers for ESCC [29]. Although *HOXA9*, a member of the *HOX* gene family, is implicated in various malignancies, its specific inclusion in methylation panels for cervical or esophageal cancer is less well documented. Nonetheless, other *HOX* family members, such as *HOXC10*, have been reported as hypermethylated in ESCC, supporting the broader relevance of *HOX* genes in cancer epigenetics [29]. While both *PAX1* and *HOXA9* (or related *HOX* genes) have been studied in the context of cancer-related methylation, their presence in the same diagnostic panels does not imply direct functional cooperation between them. Their co-involvement likely reflects parallel roles in tumor suppressor pathways affected by epigenetic dysregulation, rather than a shared mechanistic interaction.

The potential use of *SOX1* and *HOXA9* methylation as biomarkers for early detection, prognosis, and risk stratification in NSCLC is promising, but requires further exploration.

Recent advances in blood-based DNA methylation assays offer promising improvements in early lung cancer detection. A study found that a methylation-based risk score performed similarly to the PLCOm2012 model, and combining both enhanced predictive accuracy—highlighting the potential of methylation markers to strengthen existing screening strategies. Additionally, an assay in development, Lung EpiCheck^®^, which includes markers such as *HOXA9*, has demonstrated strong performance in detecting lung cancer, including early-stage disease, across European and Chinese high-risk populations, supporting its utility as a non-invasive screening tool [30,31,32,33]. Efforts should be made to investigate the influence of demographic and lifestyle factors on methylation patterns to better understand their prognostic value. Future research should also focus on developing reliable, non-invasive methods for detecting methylation changes, which could facilitate the use of these biomarkers in routine clinical practice. Integrating methylation data with other molecular and clinical parameters could lead to more comprehensive and personalized approaches to NSCLC management. By addressing and bridging these gaps, we could aspire to realize the full potential of methylation biomarkers in improving outcomes for NSCLC patients.

### Limitations

While our study provides valuable insights, it is limited by its retrospective nature and the relatively small sample size. Larger, prospective studies are needed to validate our findings and explore the mechanisms driving gene hypermethylation in NSCLC. Current research predominantly involves studies from a single country, which might limit the generalizability of findings to other populations. External validation using larger, multicenter datasets and cohorts from diverse geographic regions is essential to confirm the applicability of the identified methylation patterns in broader NSCLC populations.

Furthermore, in our study, detailed data on comorbidities were not systematically recorded for the patient cohort, which limits our ability to analyze their potential impact on survival outcomes.

Understanding the relationship between *SOX1* and *HOXA9* methylation and their expression levels would provide valuable insights. Unfortunately, gene expression data for the analyzed tissue and blood samples were not available for this study. We acknowledge this as a limitation, and we plan to incorporate gene expression analyses in future studies to better elucidate the functional impacts of *SOX1* and *HOXA9* methylation. This will allow us to draw more comprehensive conclusions about the implications of our findings.

Gene expression data for the analyzed tissue and blood samples were not available in this study. Future research integrating methylation and mRNA expression analyses is necessary to validate the functional significance of promoter hypermethylation in SOX1 and *HOXA9*.

The use of bisulfite pyrosequencing, while highly sensitive and specific, is limited to analyzing preselected CpG sites, and does not provide a genome-wide perspective on methylation changes. Advanced methods such as whole-genome bisulfite sequencing or targeted methylation panels could offer broader insights into the epigenetic landscape of NSCLC and identify additional clinically relevant methylation targets. Additionally, experimental research is needed to elucidate the detailed mechanisms by which *HOXA9* and *SOX1* methylation influence cancer progression, and to explore its potential as a therapeutic target. There is still a need for more consistent criteria for assessing *HOXA9* and *SOX1* methylation levels across studies.

Our study did not include molecular testing for *EGFR, ALK,* or *ROS1* mutations, as these analyses were not routinely performed at the time of sample collection. This limits our ability to assess the potential interactions between driver mutations and promoter hypermethylation. Future studies should incorporate comprehensive genomic and epigenomic profiling to provide a more integrated view of tumor biology and prognosis.

## Figures and Tables

**Table 1:** Analysis of hypermethylation tumor profiles (*p*-values calculated by Fisher exact test) based on patients’ clinical–pathological characteristics.

	SOX1		HOXA9	
Patients characteristics	Hypermet	Nonmet	Hypermet	Nonmet
Gender				
Male	15 (71.4)	27 (64.3)	29 (74.6)	13 (54.2)
Female	6 (28.6)	15 (35.7)	10 (25.6)	11 (45.8)
*p*-value	0.78		0.11	
Histology				
Squamocell	14 (66.7)	20 (47.6)	24 (61.5)	9 (37.5)
AdenoCa	7 (33.3)	13 (31.0)	13 (33.3)	7 (29.2)
Other	0 (0.0)	9 (21.4)	2 (5.1)	8 (33.3)
*p*-value	0.05		0.01	
Grade				
<2	9 (42.9)	12 (30.0)	13 (33.3)	8 (33.3)
2	5 (19.0)	18 (45.0)	15 (38.5)	8 (33.3)
>2	9 (38.1)	10 (25.0)	11 (28.2)	8 (33.3)
*p*-value	0.14		0.80	
Stage				
I	2 (9.5)	13 (31.7)	5 (12.8)	10 (43.5)
II	10 (47.6)	14 (34.1)	17 (43.6)	7 (30.4)
III	7 (33.3)	12 (29.3)	15 (38.5)	4 (17.4)
IV	2 (4.9)	2 (4.9)	2 (5.1)	2 (8.7)
*p*-value	0.24		0.03	
Smoking status				
Ex smoker/nonsmoker	6 (28.6)	6 (14.6)	9 (23.7)	3 (12.5)
Smoker	15 (71.4)	35 (85.4)	29 (76.3)	21 (87.5)
*p*-value	0.31		0.34	
Pack/years				
<40	4 (20.0)	14 (34.1)	9 (24.3)	9 (37.5)
40–70	12 (60.0)	19 (46.3)	22 (59.5)	9 (37.5)
>70	4 (20.0)	8 (19.5)	6 (16.2)	6 (25.0)
*p*-value	0.58		0.26	
Age				
<56	6 (28.6)	9 (21.4)	8 (20.5)	7 (29.2)
56–61	5 (23.8)	17 (40.5)	13 (33.3)	8 (33.3)
62–66	6 (28.6)	8 (19.0)	10 (25.6)	5 (20.8)
>66	4 (19.0)	8 (19.0)	8 (20.5)	4 (16.7)
*p*-value	0.57		0.90	

**Table 2:** Number of CpG islands inside promoters of genes *SOX1* and HOXA9.

Gene	CpG Islands
SOX1	4
HOXA9	11

**Table 3:** Values of measured methylation in blood, tumor and lung tissue outside the tumor at all CpG islands in both genes.

	Blood				Normal Lung Tissue				Tumor			
Gene	N	Median	Mean	StD	N	Median	Mean	StD	N	Median	Mean	StD
SOX1												
Average methylation	50	12.67	12.76	2.93	64	11.80	12.40	5.75	63	25.61	28.86	16.48
CpG1	50	9.39	9.87	3.36	64	8.36	9.84	5.73	63	21.89	25.91	18.12
CpG2	50	18.74	19.12	7.14	64	16.52	18.06	8.45	63	31.61	33.80	17.86
CpG3	50	12.28	12.16	3.58	64	10.58	11.03	6.58	63	21.60	28.19	16.70
CpG4	50	9.69	9.87	3.24	64	9.45	10.68	6.56	63	21.16	27.52	18.90
	**Blood**				**Normal lung tissue**				**Tumor**			
**Gene**	**N**	**Median**	**Mean**	**StD**	**N**	**Median**	**Mean**	**StD**	**N**	**Median**	**Mean**	**StD**
HOXA9												
Average methylation	50	3.21	3.57	1.66	64	9.96	11.44	4.80	63	32.85	32.34	16.79
CpG1	50	3.10	3.34	1.44	64	17.54	17.59	7.52	63	34.49	34.98	17.52
CpG2	50	2.95	3.17	1.37	64	15.45	15.61	4.90	63	35.97	35.22	17.52
CpG3	50	2.84	3.35	1.75	64	10.45	12.05	5.23	63	33.79	32.95	16.62
CpG4	50	2.58	3.06	1.92	64	6.31	9.11	6.12	63	34.71	32.59	18.26
CpG5	50	3.50	4.59	5.68	64	6.52	8.75	5.76	63	32.54	31.17	16.77
CpG6	50	3.10	3.34	1.26	64	9.56	10.38	4.55	63	32.31	30.86	16.24
CpG7	50	3.79	4.36	3.44	64	10.03	10.80	4.65	63	32.48	31.92	17.48
CpG8	50	2.48	2.88	1.89	64	7.13	9.06	5.24	63	29.23	28.01	14.77
CpG9	50	3.19	3.61	2.07	64	6.94	9.69	6.20	63	33.56	32.40	17.22
CpG10	50	3.48	3.91	1.84	64	10.17	12.96	8.17	63	33.53	32.98	16.94
CpG11	50	3.21	3.63	1.56	61	7.95	9.95	5.91	62	33.86	32.83	17.63

**Table 4:** *p*-values from the Wilcoxon test for the detection of statistically important differences in methylation between different types of tissue (tumor, normal lung tissue, blood). The calculation is based on mean values of all analyzed CpG islands inside both genes.

Gene	Tumor vs. Normal	Tumor vs. Blood	Normal vs. Blood
SOX1	<0.001	<0.001	0.50
HOXA9	<0.001	<0.001	<0.001

**Table 5:** ICC values for all tissue types.

Gene	ICC		
	Tumor	Normal	Blood
SOX1	0.72	0.50	0.38
HOXA9	0.96	0.74	0.67

**Table 6:** Mean values of DNA methylation for both genes (average value of all CpG islands).

Gene	Tumor			Normal Lung Tissue			Blood		
	N	Median (IQR)	Mean (SD)	N	Median (IQR)	Mean (SD)	N	Median (IQR)	Mean (SD)
SOX1	63	25.6 (15.4–36.3)	28.9 (16.5)	64	11.8 (8.9–14.1)	12.4 (5.8)	50	12.7 (11.5–14.5)	12.8 (2.9)
HOXA9	63	32.9 (19.0–45.0)	32.3 (16.8)	64	10.0 (8.4–13.1)	11.4 (4.8)	50	3.2 (2.6–4.0)	3.6 (1.7)

**Table 7:** Proportion of hypermethylation * in all tissue types in both genes.

	Tumor		Normal		Blood	
Gene	N	Freq (%)	N	Freq (%)	N	Freq (%)
*SOX1*	63	21 (33.3)	64	1 (1.6)	50	0 (0.0)
*HOXA9*	63	39 (61.9)	64	3 (4.7)	50	0 (0.0)

* More than 3SD of average value in normal lung tissue for at least 2 CpG islands.

**Table 8:** Total number of patients with hypermethylation of both genes in tumor DNA. In the table are the mean values of DNA methylation (for all CpG islands in the promotor) in both genes in tumor DNA.

	Hypermethylated						Nonhypermethylated					
Gene	N	Median	Mean	StD	Min	Max	N	Median	Mean	StD	Min	Max
*SOX1*	21	46.72	47.46	13.80	29.54	78.21	42	18.21	19.56	7.23	7.3	36.25
*HOXA9*	39	41.73	42.98	11.48	24.30	73.38	24	14.58	15.05	6.23	2.08	24.11

## Data Availability

The data presented in this study are available on request from the corresponding author.

## References

[1] Siegel R.L., Giaquinto A.N., Jemal A. (2024). Cancer statistics, 2024. CA A Cancer J. Clin..

[2] Knight S.B., Phil A., Crosbie P.A., Balata H., Chudziak J., Hussell T., Dive C. (2017). Progress and prospects of early detection in lung cancer. Open Biol..

[3] West H., Hu X., Chirovsky D., Walker M.S., Wang Y., Kaushiva A., Tepsick J., Samkari A. (2023). Clinical and economic impact of recurrence in early-stage non-small-cell lung cancer following complete resection. Future Oncol..

[4] Locke W.J., Guanzon D., Ma C., Liew Y.J., Duesing K.R., Fung K.Y., Ross J.P. (2019). DNA Methylation Cancer Biomarkers: Translation to the Clinic. Front. Genet..

[5] Saito K., Kawakami K., Matsumoto I., Oda M., Watanabe G., Minamoto T. (2010). Long interspersed nuclear element 1 hypomethylation is a marker of poor prognosis in stage IA non-small cell lung cancer. Clin. Cancer Res..

[6] Gao H., Yang J., He L., Wang W., Liu Y., Hu Y., Ge M., Ding J., Ye Q. (2022). The Diagnostic Potential of SHOX2 and RASSF1A DNA Methylation in Early Lung Adenocarcinoma. Front. Oncol..

[7] Hu H., Zhou Y., Zhang M., Ding R. (2019). Prognostic value of RASSF1A methylation status in non-small cell lung cancer (NSCLC) patients: A meta-analysis of prospective studies. Biomarkers.

[8] Nunes S.P., Diniz F., Moreira-Barbosa C., Constâncio V., Silva A.V., Oliveira J., Soares M., Paulino S., Cunha A.L., Rodrigues J. (2019). Subtyping Lung Cancer Using DNA Methylation in Liquid Biopsies. J. Clin. Med..

[9] Kontic M., Stojsic J., Jovanovic D., Bunjevacki V., Ognjanovic S., Kuriger J., Puumala S., Nelson H.H. (2011). Aberrant promoter methylation of CDH13 and MGMT genes is associated with clinicopathological characteristics of primary non small cell lung carcinoma. Clin. Lung Cancer.

[10] Hoang P.H., Landi M.T. (2022). DNA Methylation in Lung Cancer: Mechanisms and Associations with Histological Subtypes, Molecular Alterations, and Major Epidemiological Factors. Cancers.

[11] Kontic M., Jovanovic D., Kern I., Nelson H.H., Bojic S., Ognjanovic M., Ognjanovic S. (2022). Is hypermethylation of SOX1 gene an independent prognostic marker in surgically resected non-small cell lung cancer?. J. Cancer Res. Ther..

[12] Abou-Zeid A., Hashad D., Baess A., Mosaad M., Tayae E. (2023). HOXA9 gene promotor methylation and copy number variation of SOX2 and HV2 genes in cell free DNA: A potential diagnostic panel for non-small cell lung cancer. BMC Cancer.

[13] Chang W.L., Pan M.J. (1996). Specific amplification of Ehrlichia platys DNA from blood specimens by two-step PCR. Clin. Microbiol..

[14] Brock M.V., Hooker C.M., Ota-Machida E., Han Y., Guo M., Ames S., Glöckner S., Piantadosi S., Gabrielson E., Pridham G. (2008). DNA methylation markers and early recurrence in stage I lung cancer. N. Engl. J. Med..

[15] Nelson H.H., Marsit C.J., Christensen B.C., Houseman E.A., Kontic M., Wiemels J.L., Karagas M.R., Wrensch M.R., Zheng S., Wiencke J.K. (2012). Key epigenetic changes associated with lung cancer development: Results from dense methylation array profiling. Epigenetics.

[16] Shames D.S., Minna J.D., Gazdar A.F. (2007). Methods for detecting DNA methylation in tumors: From bench to bedside. Cancer Lett..

[17] Ooki A., Maleki Z., Tsay J.-C.J., Goparaju C., Brait M., Turaga N., Nam H.-S., Rom W.N., Pass H.I., Sidransky D. (2017). A Panel of Novel Detection and Prognostic Methylated DNA Markers in Primary Non-Small Cell Lung Cancer and Serum DNA. Clin. Cancer Res..

[18] Wen S.W.C., Andersen R.F., Petersen L.M.S., Hager H., Hilberg O., Jakobsen A., Hansen T.F. (2020). Comparison of Mutated KRAS and Methylated HOXA9 Tumor-Specific DNA in Advanced Lung Adenocarcinoma. Cancers.

[19] Cai H., Ke Z.-B., Dong R.-N., Chen H., Lin F., Zheng W.-C., Zhu J.-M., Chen S.-M., Zheng Q.-S., Wei Y. (2021). The prognostic value of homeobox A9 (HOXA9) methylation in solid tumors: A systematic review and meta-analysis. Transl. Cancer Res..

[20] Hwang J., Bin Lee B., Kim Y., Hong S., Kim Y., Han J., Shim Y.M., Yoon C., Lee Y., Kim D. (2015). *HOXA9* inhibits migration of lung cancer cells and its hypermethylation is associated with recurrence in non-small cell lung cancer. Mol. Carcinog..

[21] Li N., Li S. (2015). Epigenetic inactivation of SOX1 promotes cell migration in lung cancer. Tumor Biol..

[22] Wen S.W.C., Nederby L., Andersen R.F., Nyhus C.H., Hilberg O., Jakobsen A., Hansen T.F. (2023). NK cell activity and methylated HOXA9 ctDNA as prognostic biomarkers in patients with non-small cell lung cancer treated with PD-1/PD-L1 inhibitors. Br. J. Cancer.

[23] Vicente A.L.S.A., Santos F.A.d.S., Hirai W.Y., Lissa D., Cavagna R.d.O., da Silva A.L.V., dos Reis M.B., da Silva E.C.A., da Silva F.A.F., Mourão J.D. (2025). HOXA9 methylation is not associated with survival in Brazilian patients with lung adenocarcinoma. Clin. Epigenetics.

[24] Toyooka S., Suzuki M., Maruyama R., O Toyooka K., Tsukuda K., Fukuyama Y., Iizasa T., Aoe M., Date H., Fujisawa T. (2004). The relationship between aberrant methylation and survival in non-small-cell lung cancers. Br. J. Cancer.

[25] Vaissière T., Hung R.J., Zaridze D., Moukeria A., Cuenin C., Fasolo V., Ferro G., Paliwal A., Hainaut P., Brennan P. (2009). Quantitative analysis of DNA methylation profiles in lung cancer identifies aberrant DNA methylation of specific genes and its association with gender and cancer risk factors. Cancer Res..

[26] Du C., Tan L., Xiao X., Xin B., Xiong H., Zhang Y., Ke Z., Yin J. (2024). Detection of the DNA methylation of seven genes contribute to the early diagnosis of lung cancer. J. Cancer Res. Clin. Oncol..

[27] Bhatlekar S., Viswanathan V., Fields J.Z., Boman B.M. (2018). Overexpression of HOXA4 and HOXA9 genes promotes self-renewal and contributes to colon cancer stem cell overpopulation. J. Cell. Physiol..

[28] Talarmain L., Clarke M.A., Shorthouse D., Cabrera-Cosme L., Kent D.G., Fisher J., Hall B.A. (2022). *HOXA9* has the hallmarks of a biological switch with implications in blood cancers. Nat. Commun..

[29] Gao Y., Zi D., Liang W., Qiu F., Zheng J., Xiao X., Jiang E., Xu Y. (2024). PAX1 and SOX1 Gene Methylation as a Detection and Triage Method for Cervical Intraepithelial Neoplasia Diagnosis. Acta Cytol..

[30] Lai H.C., Ou Y.-C., Chen T.-C., Huang H.-J., Cheng Y.-M., Chen C.-H., Chu T.-Y., Hsu S.-T., Liu C.-B., Hung Y.-C. (2014). PAX1/SOX1 DNA methylation and cervical neoplasia detection: A Taiwanese Gynecologic Oncology Group (TGOG) study. Cancer Med..

[31] Yu Q., Xia N., Zhao Y., Jin H., Chen R., Ye F., Chen L., Xie Y., Wan K., Zhou J. (2022). Genome-wide methylation profiling identify hypermethylated HOXL subclass genes as potential markers for esophageal squamous cell carcinoma detection. BMC Med. Genom..

[32] Xu Y., Wang Z., Pei B., Wang J., Xue Y., Zhao G. (2024). DNA methylation markers in esophageal cancer. Front. Genet..

[33] Gaga M., Chorostowska-Wynimko J., Horváth I., Tammemagi M.C., Shitrit D., Eisenberg V.H., Liang H., Stav D., Faber D.L., Jansen M. (2021). Validation of Lung EpiCheck, a novel methylation-based blood assay, for the detection of lung cancer in European and Chinese high-risk individuals. Eur. Respir. J..

